# Anterior cervical microforaminotomy for persistent brachialgia in a patient with multilevel cervical spondylosis; comparing PROMIS with Nurick score for outcome of surgery

**DOI:** 10.1016/j.ijscr.2021.106400

**Published:** 2021-09-13

**Authors:** Muhtamim Chowdhury, Md Moshiur Rahman

**Affiliations:** a Neurosurgery Department, Enam Medical College, Dhaka, Bangladesh; b Neurosurgery Department, Holy Family Red Crescent Medical College, Dhaka, Bangladesh

**Keywords:** Cervical microforaminotomy, Brachialgia, Multilevel cervical spondylosis

## Abstract

**Introduction and importance:**

The primal instinct of neurosurgeons has been to maintain spinal stability and motion since the beginning of spinal procedures. Conventional anterior approaches without fusion eliminate motion in time as fusion invariably sets in and hampers the vertebral column's normal dynamic physiology.

**Case presentation:**

We reported a 60 years old male patient who presented with signs of myelopathy, but his primary complaint was brachialgia. He had myelopathic features for eight years, for which he offered fusion surgery at multiple levels years ago, and he denied it. He was static since then, and the disease did not progress further. For intolerable pain, he agreed to minimally invasive surgery. Therefore, we operated for a right C6 transcorporeal microforaminotomy and removed the inciting disc material.

**Clinical discussion:**

Clinical implication for anterior cervical microforaminotomy for this patient was successful where there was acute disc prolapse in cervical spondylotic myelopathy.

**Conclusion:**

Finally, functional preservation of the cervical spine in multilevel spondylosis can optimize the fusion. In this case, the report authors have explored the comparison between the PROMIS score and the Nurick score, reporting for the first time.

## Highlights

• Functional retention of cervical spine can boost fusion in multilevel spondylosis.
• Clinical efficacy of cervical microforaminotomy in acute disc prolapse is effective.
• The case report shows comparison of PROMIS score and Nurick score for the first time.

## 1 Introduction

Since the inception of spinal procedures, it has been the primal instinct of neurosurgeons to maintain spinal stability and preserve its motion. Mixter and Barr first described pure cervical radiculopathy around 1934 and then published a case series of 19 patients treated with posterior cervical laminectomy [1]. Since then, cervical radiculopathy has received wide attention ranging from posterior keyhole laminotomy with or without discectomy to various anterior approaches. First, Robinson and Smith and then later Cloward in 1958 devised and paved the pathway to modernize anterior techniques [2], [3]. Even though posterior approaches nowadays are reserved for the spondylotic myelopathies, it is used by many surgeons in treating cervical radiculopathies. But the challenge remains in addressing the issues placed anteriorly. Thus Jho et al. in 1996 introduced or, better say, refined the anterior cervical foraminotomy for radiculopathies due to focal disc herniation [4]. Traditional anterior approaches without fusion eliminate motion in time as fusion invariably sets in and hampers the vertebral column's normal dynamic physiology. Fusion procedures further degenerative changes in adjacent segments due to loss of mobility. Patient-Reported Outcomes Measurement Information System (PROMIS), physical and mental.

## 2 Case report

**Fig. 1:**
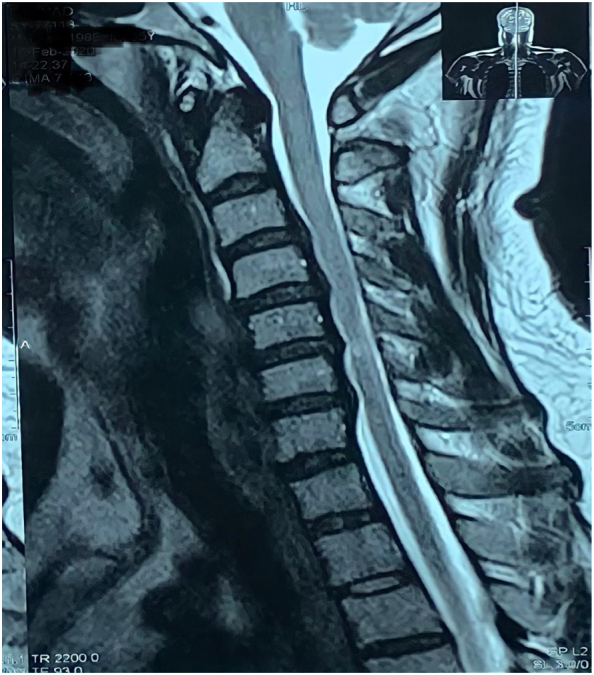
MRI of cervical spine in sagittal T2 image showing multilevel compression at C4 to C7.

**Fig. 2:**
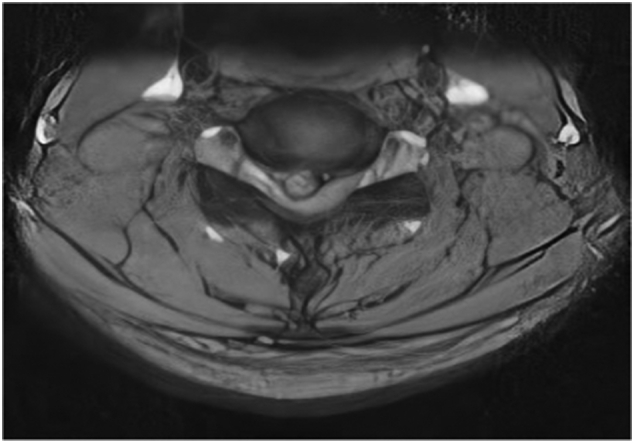
Axial T2 image revealing right-sided lateral disc prolapse.

Our patient is a 60 years old gentleman who presented with chronic nagging neck pain complaints for four years, which progressed to severe right brachialgia for the last three months. The gentleman is hypertensively controlled on medication otherwise normal. Our clinical examination revealed the right elbow extension weakness with right-sided diminished sensory to pinprick at C7 (Fig. 2). On eliciting the deep tendon reflexes (DTR), the right triceps jerk was absent. Hoffman was present on the right side but not on the left. Spurling and abductor relief sign was present on the right and Lhermitte's sign for spondylotic myelopathy. Lower limbs examination was regular with no undue spasticity or exaggerated DTR. He had myelopathic features for eight years, for which he offered fusion surgery at multiple levels years ago, and he denied it. He was static since then, and the disease did not progress further. For intolerable pain, he agreed to minimally invasive surgery. On radiological evaluation, MRI cervical spine showed multilevel disc degeneration, T2 hyperintense signal abnormality with prolapse, particularly a lateral herniation on the suitable C7 exiting root (Fig. 1). There were also multiple posterior osteophytic spurs, compression segmentally on the cord, and hampering the CSF flow.

**Fig. 3:**
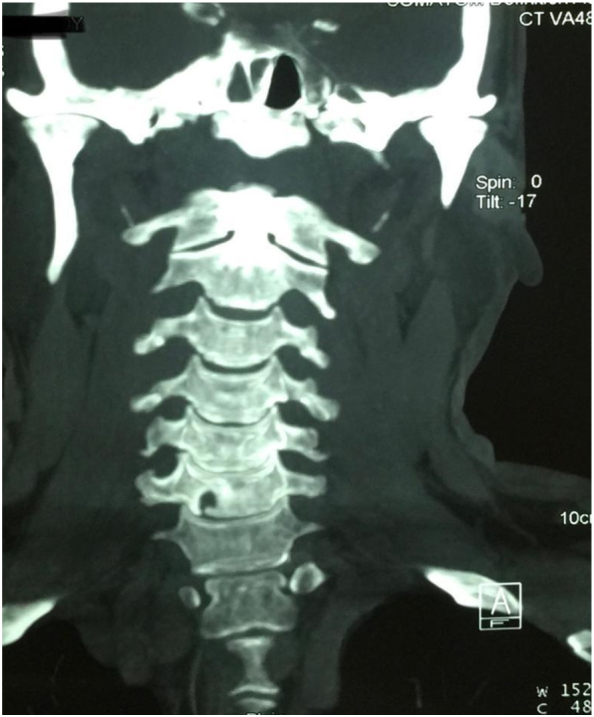
CT scan of cervical spine showing drill hole at C6 for C6/7 right lateral disc herniation.

**Fig. 4:**
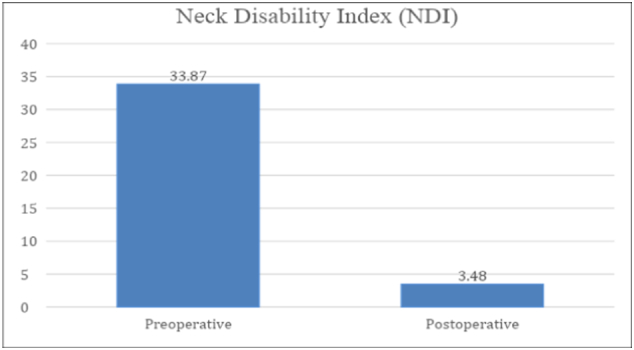
Preoperative and postoperative NDI score of the patient.

**Fig. 5:**
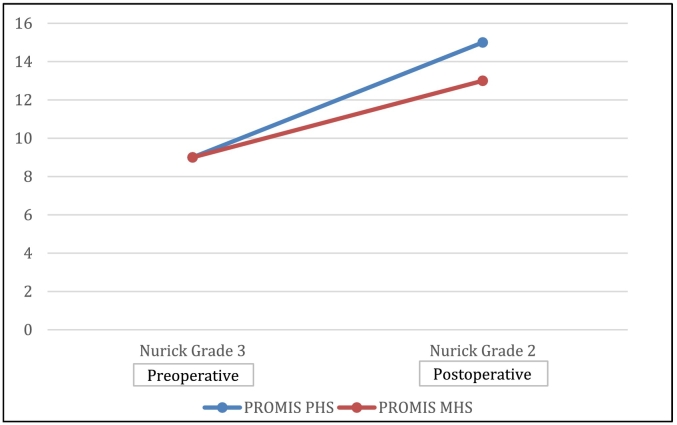
Comparison of health status according to PROMIS and Nurick scoring.

We found electrophysiological tests EMG and NCS are consistent with cervical spondylosis. Clinically, even though he presented with signs of myelopathy, his primary complaint was brachialgia. Here in this particular case, we found the myelopathy was static with no deterioration after eight years. So, we opted for a right C6 transcorporeal microforaminotomy and removed the inciting disc material. The work has been reported in line with the SCARE 2020 considerations [5].

## 3 Operative technique

The position is similar to any other anterior procedure, with the neck slightly extended and head fixed with a halter traction strap. But unlike traditional ACDF, the side of the approach is ipsilateral to the side of the radiation. I prefer the transverse incision and a made one level higher than the affected disc herniation. After the usual sharp and blunt fascial plan dissection and the self-retaining retractor set placement, the C-arm ensured the operational level. The longus colli was then sequentially dissected off one level above and below that; the retractor system is repositioned in front of it.

Using the high-speed drill set and appropriate burr, a primary 5–6 mm hole is made, 4–5 mm above the lower vertebral border at the upper vertebra's inferolateral portion (Fig. 3). The drilling direction is medial to lateral, but care must be taken not to open up the medial wall of the foramen transversarium and unduly expose the ipsilateral vertebral artery. The posterior longitudinal ligament is breached using a micro hook, and the extruded portion of the disc is removed using either a microhook or micro rongeur. The decompression's adequacy is assessed against the freeness of the microhook within the epidural space, palpating the pedicle, and the CSF pulsation at the end of the procedure. Then the wound was closed in layers.

The patient was followed up at one month, six months, one year, and 2 years at the hospital, and the outcome of surgery was measured in the hospital at 2 years which is stated below.

The Neck Disability Index (NDI) scores were assessed in the patient, showing that his condition improved gradually (Fig. 4). His preoperative and postoperative NDI score was 33.87 and 3.48 respectively.

We assessed the patient's pre and postoperative physical and mental health status with PROMIS (Patient-Reported Outcomes Measurement Information System) Global-10 scoring system (Fig. 5). We followed the PROMIS questionnaire and calculated his scores, which were 9 points preoperatively and postoperatively 15 points (physical health score, PHS) and 13 points (mental health score, MHS), respectively. In addition, he was assessed according to the Nurick grade score (Grade 0-Grade 4). Preoperatively he was at grade 3 and postoperatively at grade 2.

Both these scores are helpful within the separate ambit of functionalities.

PROMIS score highlighted the comprehensive scoring in which we can assess the details of patients' matters.

## 4 Discussion

According to many authors, patient identification to justify the anterior trans-corporeal approach is of utmost importance [1]. In our opinion, patients with unilateral radiculopathy up to two levels are the best candidates for such procedures.

Anecdotally cervical approaches began with posterior routes initially but slowly fell out of favor, even though continued by a few. The inability to work in front of the affected nerve root is the main difficulty to address brachialgia due to disc fragments [2]. According to Robinson and Smith, and Cloward in 1958, anterior cervical approaches began to take fashion [2], [3]. But both these techniques resulted in a fusion of the involved elements, which was not a physiological treatment, some argued. Even when fusion was not carried out, results with fusion were comparable, and ultimately, fusion did eliminate a mobile segment.

Verbiest and Hakuba sequentially introduced anterolateral techniques in 1968 and 1976 [6]. Both the procedures were very radical, involving the Longus Colli's transection to gain access to the transverse process and completely denuding the Vertebral Artery by removing the ipsilateral uncovertebral joint. Later smaller studies by Lesoin et al. and Snyder and Bernhardt were more refined and brachialgia directed compared to their predecessors but both procedures involved fusion of the surgical segments of interest [7], [8]. Our technique, which follows the principles detailed by Jho in 1996, doesn't involve completely removing the intervertebral disc and rendering the segment unfunctional due to subsequent fusion [4].

Oshima et al. discovered that surgery in signal intensity changes in MRI in patients with myelopathy is not obligatory for surgery [10]. At the same time, Vedantam et al. and other authors found it to end with poor outcomes after surgery in T2 weighted signal intensity patients with cervical myelopathy [11], [12].

Jho, in 1996 presented the first study detailing surgical anatomy, technique, and outcome [4]. Later Johnson et al. similarly presented a case series of 21 patients and substantiated Jho's evidence [9]. We operated on the same principle of Jho with the primary goal to preserve the motion of the involved segment. The Anterior Transcorporeal approach's single-level brachialgia has the added advantage of being a shorter, anatomy and physiology-preserving surgery [1].

## 5 Conclusion

We made a point by considering that clinicians should address clinical judgment, not the radiological one. Functional preservation of the cervical spine in multilevel spondylosis can optimize the fusion. Surgical outcome comparing the PROMIS and Nurich score is justified even though a large series is needed for the conclusion. Clinical implication for anterior cervical microforaminotomy for this patient was successful with acute disc prolapse in static cervical spondylotic myelopathy. Finally, the patient was satisfied with the approach and effort made by the medical team. The present case reports for the first time shows comparison between the PROMIS score and the Nurick score in such cases.

## Informed consent

Written informed consent was obtained from the patient for publication of this case report and accompanying images. A copy of the written consent is available for review by the Editor-in-Chief of this journal on request.

## Provenance and peer review

Not commissioned, externally peer-reviewed.

## Ethical approval

Ethical approval was taken from the Ethics Review Committee of Bangladesh Bioethics Society (ERC/BBS/025).

## Funding

None.

## Guarantor

Md Moshiur Rahman.

## Research registration number

Not applicable.

## CRediT authorship contribution statement

All authors equally contributed to the analysis and writing of the manuscript.

## Declaration of competing interest

All authors declare that there exist no commercial or financial relationships that could, in any way, lead to a potential conflict of interest.
